# Axitinib in Ponatinib-Resistant B-Cell Acute Lymphoblastic Leukemia Harboring a T315L Mutation

**DOI:** 10.3390/ijms21249724

**Published:** 2020-12-20

**Authors:** Valentina Giudice, Andrea Ghelli Luserna di Rorà, Bianca Serio, Roberto Guariglia, Maria Benedetta Giannini, Anna Ferrari, Giorgia Simonetti, Carmine Selleri, Giovanni Martinelli

**Affiliations:** 1 Department of Medicine, Surgery and Dentistry “Scuola Medica Salernitana”, University of Salerno, Baronissi, 84081 Salerno, Italy; cselleri@unisa.it; 2 Clinical Pharmacology, University Hospital “San Giovanni di Dio e Ruggi D’Aragona”, 84131 Salerno, Italy; 3 Hematology and Transplant Center, University Hospital “San Giovanni di Dio e Ruggi D’Aragona”, 84131 Salerno, Italy; bianca.serio@sangiovannieruggi.it (B.S.); roberto.guariglia@sangiovannieruggi.it (R.G.); 4 Biosciences Laboratory, Istituto Scientifico Romagnolo per lo Studio e la Cura dei Tumori (IRST) IRCCS, 47014 Meldola, Italy; anna.ferrari@irst.emr.it (A.F.); giorgia.simonetti@irst.emr.it (G.S.); 5 Hematology Unit, Istituto Scientifico Romagnolo per lo Studio e la Cura dei Tumori (IRST) IRCCS, 47014 Meldola, Italy; mariabenedetta.giannini@irst.emr.it; 6 Scientific Directorate, Istituto Scientifico Romagnolo per lo Studio e la Cura dei Tumori (IRST) IRCCS, 47014 Meldola, Italy; giovanni.martinelli@irst.emr.it

**Keywords:** axitinib, ponatinib, TKI inhibitor, acute lymphoblastic leukemia

## Abstract

Adult acute lymphoblastic leukemia (ALL) with BCR-ABL1 rearrangement (Philadelphia chromosome, Ph) is a hematological aggressive disease with a fatal outcome in more than 50% of cases. Tyrosine kinase inhibitors (TKIs) targeting the activity of BCR-ABL1 protein have improved the prognosis; however, relapses are frequent because of acquired somatic mutations in the BCR-ABL1 kinase domain causing resistance to first, second and third generation TKIs. Axitinib has shown in vitro and ex vivo activity in blocking ABL1; however, clinical trials exploring its efficacy in ALL are missing. Here, we presented a 77-year-old male with a diagnosis of Ph positive ALL resistant to ponatinib and carrying a rare threonine to leucine (T315L) mutation on *BCR-ABL1* gene. The patient was treated with axitinib at 5 mg/twice daily as salvage therapy showing an immediate although transient benefit with an overall survival of 9.3 months. Further dose-finding and randomized clinical trials are required to assess the real efficacy of axitinib for adult Ph positive ALL resistant to third generation TKIs.

## 1. Introduction

Acute lymphoblastic leukemia (ALL), a malignant hematological disease mostly affecting children aged 2–4 years and adults over 50 years, is characterized by clonal expansion of T or B cell precursors harboring specific genetic abnormalities with different prognostic values [1,2]. Only 30–40% of adult ALL patients achieve a long-term complete remission, due to a lower tolerability of high dose induction chemotherapy compared with younger patients and an increased frequency of genetic alterations with poor prognostic values, such as *BCR-ABL1* rearrangement (Philadelphia chromosome, Ph) [1,2,3,4]. Indeed, among B-ALL, the Ph-positive sub-group is characterized by the worst prognosis (5-year survival of 5–46%), and prevalence of t (9;22) increases with age [1]. The introduction of tyrosine kinase inhibitors (TKIs) targeting the activity of BCR-ABL1 fusion protein has significantly improved clinical outcome of Ph-positive ALL with a probability of one-year survival of 74% also in patients older than 60 years [5,6,7,8]. However, more than 70% of Ph-positive patients, and especially those who are maintained with a single agent TKI, can develop point mutations in the *BCR-ABL1* kinase domain, and the threonine to isoleucine mutation at codon 315 (T315I) is the most frequent one, causing resistance to first and second generation TKIs [9]. Ponatinib, a third generation TKI has shown clinical efficacy in treatment of resistant Ph-positive ALL especially patients carrying the T315I mutation [10].

## 2. Case Presentation

**Figure 1:**
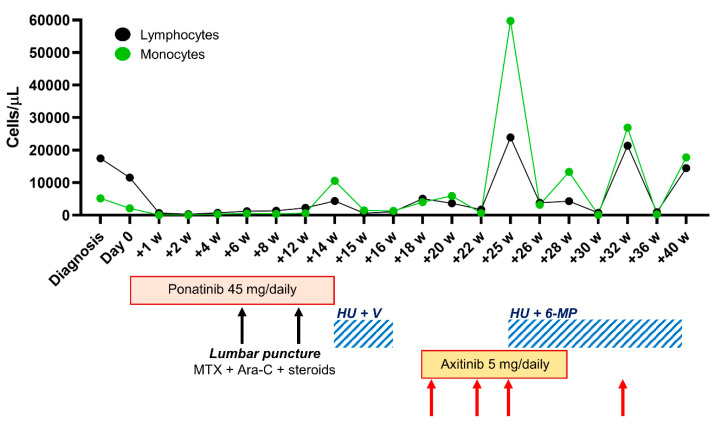
Clinical course of our ponatinib-resistant acute lymphoblastic leukemia (ALL) patient. Lymphocyte (black) and monocyte (green line) counts are reported from diagnosis to death (w, week). Type and duration of each treatment are reported: ponatinib 45 mg/daily from day 0 to +14 w; hydroxyurea (HU) 500 mg/twice daily and vincristine (V) 2 mg/weekly (light blue dashed square) from +14 w to +16 w; axitinib 5 mg/daily from +18 w to +28 w; HU 500 mg/twice daily and mercaptopurine (6-MP) 50 mg/daily based on CBC from +25 w until death. Lumbar puncture (black arrows) was performed at +6 w and +12 w with methotrexate (MTX) 10 mg, cytarabine (Ara-C) 40 mg, and steroids 4 mg. Vincristine infusion (red arrows) at 2 mg was given on weeks +19, +22, +25, and +32.

We report a 77-year-old male admitted to the hospital for fatigue, malaise, and arrhythmia with a history of hypertension and prostate cancer surgically removed ten years earlier. At baseline, complete blood counts (CBC) showed an increased number of lymphocytes (17,470 cells/µL) and anemia (8.3 g/dL) thus requiring red blood cell transfusion (Figure 1). The patient presented mild hepatosplenomegaly, and mild aortic valve stenosis. Bone marrow (BM) aspiration displayed increased frequency of lymphocytes positive for CD45, CD34, CD19, CD10, CD20, CD38, and Tdt by flow cytometry. Since the neoplastic clone harbored the Ph type P190 rearrangement, documented by RT-PCR and fluorescence in situ hybridization analysis, the patient was enrolled in the LAL 1811 GIMEMA protocol (approved by the Ethic Committee “Campania Sud” for treatment of elderly Ph-positive ALL patients) with ponatinib 45 mg/daily and steroids 20 mg/twice daily which were already administered as soon as the patient received a diagnosis of ALL.

At day 14, he was discharged because of hematological improvement and he was scheduled for weekly clinical visits. On the 5th week, he received central nervous system prophylaxis (medication with methotrexate 10 mg, cytarabine 40 mg, and steroids 4 mg), repeated on week 7 and 12. Flow cytometry analysis of BM aspiration on week 12 revealed the presence of a blast cell population accounting for 14% of total mononucleated cells indicating disease progression confirmed by RT-PCR (BCR-ABL/ABL ratio of 96.23 in the BM). Sequencing analysis on both peripheral blood and BM specimens showed the presence of a T315L mutation on *BCR-ABL1* gene. Two weeks later, he had fever without chilling, chest pain during inhalation, and 10,590 monocytes on CBC. Ponatinib was stopped, and hydroxyurea 500 mg/twice daily, vincristine 2 mg/weekly and steroids 25 mg/twice daily were started. After two weeks, in the absence of any hematological improvement (lymphocytes, 5080 cells/µL; and monocytes, 4030 cells/µL), we evaluated the possibility of using axitinib as salvage therapy.

## 3. Discussion

**Figure 2:**
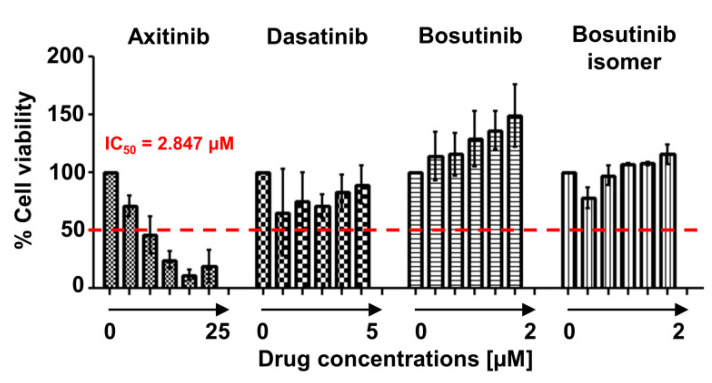
Ex vivo sensitivity of primary leukemic cells to axitinib. Patient’s peripheral blood mononuclear cells were treated with serial dilutions of axitinib (0–25 µM, *n* = 5 doses), dasatinib (0–5 µM, *n* = 5 doses), bosutinib (0–2 µM, 5 doses) and bosutinib isomer (0–2 µM, *n* = 5 doses) for 24 h at 37 °C and 5% CO_2_. Cell viability was determined by colorimetric assay and normalized on vehicle-control (DMSO-treated cells) for each drug. Axitinib showed a significant growth inhibitory effect. Data are shown as mean + SD of 3 replicates, and the inhibitory concentration 50 (IC50) for axitinib is reported.

Axitinib is a vascular endothelial growth factor receptor (VEGFR) inhibitor approved for advanced renal cell carcinoma (RCC) resistant to cytokines or other TKIs. Evidence shows that axitinib can block BCR-ABL1 kinase, and preferentially inhibits mutated autophosphorylated ABL1 compared with the wild-type protein, by binding the A-loop of the active kinase conformation without forming hydrogen bond with the T315 residue [11]. In vitro and ex vivo studies showed that axitinib is effective against T315I mutant cells, and the T315L variant represents a selective vulnerability of the drug [11,12,13]. Based on this evidence, we first tested ex vivo drug sensitivity by treating patient’s mononuclear cells with axitinib and other TKIs at various doses. Ex vivo experiments were performed on fresh peripheral blood collected right after the identification of T315L positive clones from samples collected on the 12th week when patient relapsed and ponatinib was stopped. Peripheral blood mononuclear cells were incubated with increasing concentrations of axitinib, dasatinib, bosutinib, and bosutinib isomer [14] for 24 h at 37 °C and 5% CO_2_, and cell viability was determined by colorimetric assay (WST-1, Roche). A significant growth inhibition was observed only in axitinib-treated cells, with an IC_50_ of 2.847 µM (Graph Pad Prism software, Figure 2). These results, although preliminary, supported the choice of axitinib as salvage therapy in our patient.

After informed consent obtained for off-label use approved by the Ethic Committee Drug Committee of “Area Vasta Emilia Centro” (CF AVEC; reference no. 16357 approved on 4 August 2016), the patient was treated with axitinib at 5 mg/twice daily, and hydroxyurea 500 mg/daily and/or mercaptopurine 50 mg/daily were introduced or discontinued based on CBC. Immediate clinical and hematological improvements were documented with a partial normalization of CBC (hemoglobin, 11.4 g/dL; lymphocytes, 2990 cells/µL; and 97,000 platelets/µL); however, lymphocyte count abruptly increased (23,940 cells/µL) after almost three months of treatment, and we decided to stop axitinib while hydroxyurea 500 mg/daily and/or mercaptopurine 50 mg/daily were introduced. Two months later, he showed lymphadenopathies in multiple sites by CT scan and mild splenomegaly, and he also developed a deep vein thrombosis of the left limb. The patient died one month later with an overall survival of 9.28 months and a progression-free survival of 3.24 months. Outcome of ponatinib-resistant patients in the accelerated phase of chronic myeloid leukemia is reported to be poor (overall survival of 8.9 months), and subjects are frequently refractory to subsequent therapies including TKIs and standard chemotherapy with cytarabine or decitabine [15]. Patients with ALL carrying the somatic T315I mutation have a poor prognosis that can be improved using ponatinib or CAR-T cells as salvage therapy and bridging to allogeneic hematopoietic stem cell transplantation (from 3.3 months to 13.3) [16]. However, the real impact of the T315L mutation in Ph-positive ALL is still under investigation.

The present case report describes the first case of Ph-positive ALL resistant to the third generation TKI treated with axitinib as salvage therapy. Our pre-clinical findings suggest a possible efficacy of axitinib in treatment of Ph-positive hematological disorders, especially in those patients who become resistant to other TKIs [9]. The lack of a lasting hematological improvement of the patient under axitinib treatment compared with standard chemotherapy was likely due to the low axitinib dosage that was administered (the lowest as per RCC protocol), in order to reduce the risk of unexpected side effects. However, RCC cell survival strongly relies on VEGFR pathway and low axitinib concentrations can be effective, while the inhibition of mutant BCR-ABL1 ALL is obtained at higher concentrations and leukemic cells have also several dysregulated pathways [14]. Moreover, we cannot exclude a role of vincristine and hydroxyurea on decreasing lymphocyte count; however, they probably had a minimal role as monocyte count was not significantly affected. Nevertheless, our ponatinib-resistant ALL patient treated with even low dose axitinib showed an immediate although transient clinical and hematological improvement. Therefore, further larger pre-clinical and clinical studies are required to better understand the mechanism of action of axitinib against ALL clones and to identify groups of patients who might benefit of this TKI as single agent or in combination with standard antileukemic agents.
